# Pulmonary hypertension and NT-proBNP dynamics during the course of adulticide treatment in dogs naturally infected by *Dirofilaria immitis*

**DOI:** 10.1186/s13071-025-06945-2

**Published:** 2025-07-31

**Authors:** Noelia Costa-Rodríguez, Daniel Julio Vera-Rodríguez, Soraya Falcón-Cordón, Beatriz Regina Morales, José Alberto Montoya-Alonso, Rodrigo Morchón, Elena Carretón

**Affiliations:** 1 Internal Medicine, Faculty of Veterinary Medicine, Research Institute of Biomedical and Health Sciences (IUIBS), University of Las Palmas De Gran Canaria, Las Palmas, Spain; 2 Zoonotic Diseases and One Health Group, Laboratory of Parasitology, Faculty of Pharmacy, University of Salamanca, Salamanca, Spain

**Keywords:** Heartworm, NT-proBNP, Pulmonary hypertension, Heartworm disease, Biomarker, Adulticide treatment, Canine cardiology, Echocardiography

## Abstract

**Background:**

Pulmonary hypertension (PH) is a frequent complication in dogs with heartworm disease caused by *Dirofilaria immitis*. Although echocardiography remains the main diagnostic tool, its operator- and preload-dependence may limit accuracy. N-terminal pro-B-type natriuretic peptide (NT-proBNP) is a cardiac biomarker that increases in response to ventricular wall stress and may be useful for monitoring right-sided heart disease. This study aimed to evaluate NT-proBNP concentrations in dogs with precapillary PH due to heartworm disease during adulticide treatment.

**Methods:**

In total, 90 dogs diagnosed with heartworm disease were prospectively enrolled and classified according to the presence of PH based on echocardiographic criteria. NT-proBNP concentrations were measured on days 0, 30, 60, and 90 of adulticide treatment. Additional data collected included the presence/absence of microfilariae, clinical signs, parasite burden, and renal values. Dogs received adulticidal therapy following current international guidelines. Statistical analyses assessed correlations between NT-proBNP levels, epidemiological, clinical and echocardiographic classification, and treatment progression.

**Results:**

Dogs with PH had significantly higher NT-proBNP concentrations at baseline compared with those without PH (2038 ± 1671 versus 583 ± 185 pmol/L, *P* < 0.001). NT-proBNP levels were also positively correlated with parasite burden (*r* = 0.530, *P* < 0.05), presence of clinical signs (*r* = 0.456, *P* < 0.05), and age (*r* = 0.29, *P* < 0.05). During treatment, a progressive decrease in NT-proBNP concentrations was observed in dogs with PH, while levels remained stable in dogs without PH. Receiver operating characteristic (ROC) analysis identified a cut-off of 1524.8 pmol/L for detecting moderate-to-severe PH (sensitivity: 99%, specificity: 87%).

**Conclusions:**

NT-proBNP is a valuable noninvasive biomarker for detecting and monitoring PH in dogs with heartworm disease. Its concentrations seem to reflect parasite burden, clinical status, and echocardiographic severity, and decline progressively with adulticide therapy. Integration of NT-proBNP into diagnostic and therapeutic protocols may enhance management of heartworm-infected dogs with suspected PH.

**Graphical Abstract:**

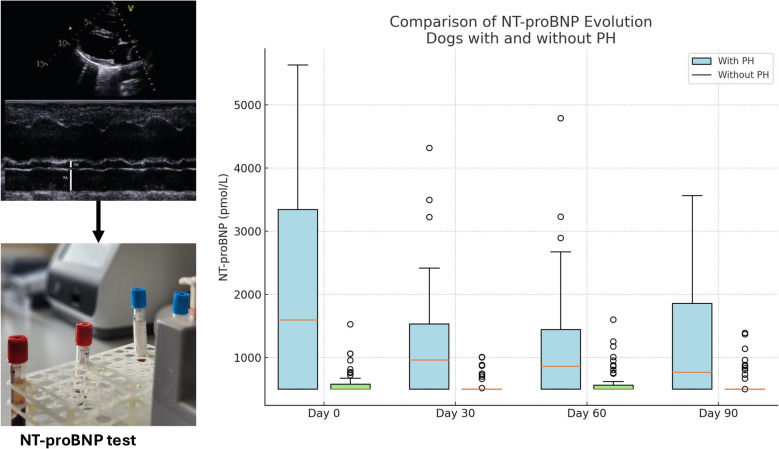

## Background

Pulmonary hypertension (PH) is defined as increased pressure within the pulmonary vasculature. It is a hemodynamic and pathophysiologic condition present in a wide variety of cardiovascular, respiratory, and systemic diseases, such as heartworm disease [1]. It has been previously reported that *Dirofilaria immitis* causes vascular changes in dogs, including proliferative endarteritis of the pulmonary arteries, which begins very soon after the arrival of the adult worms [2]. The reduction in arterial lumen, along with the loss of elasticity and compliance of the arteries, can chronically affect the arterial side of the pulmonary vascular system and is referred to as precapillary PH. If not controlled, precapillary PH can lead to right-sided congestive heart failure and become potentially life-threatening [2, 3].

The diagnosis can be challenging. The gold standard for determining PH is right heart catheterization; however, the cost and technical requirements of this direct evaluation of pulmonary arterial pressure make it a limited technique in veterinary medicine [1, 4]. Conversely, echocardiography has been demonstrated to be a viable alternative to invasive methods, particularly using transthoracic Doppler echocardiography [5]. Estimating the peak systolic pulmonary arterial pressure by measuring the tricuspid regurgitation peak velocity (TRV) and calculating the right ventricular to right atrial pressure gradient (RV:RA PG) can help assess the probability that a dog has PH [1, 6, 7]. In addition, assessment of the pulmonary regurgitation (PR) jet can offer an alternative approach for estimating mean or diastolic pulmonary arterial pressure [8–10]. When tricuspid regurgitation and/or PR are not present, the right pulmonary artery distensibility index (RPAD Index) has been validated as a valuable method to estimate the presence and severity of PH in heartworm-infected dogs [1, 11, 12].

Echocardiography is operator-dependent, requiring optimal three-dimensional alignment, and is preload-dependent, in contrast to cardiac biomarkers, whose concentrations can be measured independently of the operator using a blood test [13]. One of the most studied biomarkers is brain natriuretic peptide (BNP). In humans, it has been used in cases of precapillary PH and is recommended for initial risk stratification [13–16]. BNP exists as a prohormone that is cleaved into the inactive N-terminal fragment (NT-proBNP) and the biologically active hormone BNP prior to its release into the bloodstream in response to ventricular myocyte stretch [17]. The half-life of NT-proBNP is longer than that of the active hormone BNP, and it exhibits greater stability in circulating blood and after sampling [18–20]. Several studies in dogs have suggested that serum NT-proBNP levels can increase in the presence of precapillary PH, making this biomarker a valuable diagnostic tool when used alongside historical data, physical examination, and diagnostic imaging [21–23]. In addition, a previous study in dogs infected with *D. immitis* evaluated NT-proBNP concentrations, reporting an increase in this biomarker in dogs with precapillary PH [24].

Given the relevance of NT-proBNP as a potential biomarker for determining precapillary PH in dogs with heartworm disease, this study aimed to evaluate its response throughout adulticidal treatment in dogs infected with this parasite, with or without PH.

## Methods

### Animals studied

This prospective study included 90 dogs diagnosed with heartworm disease, all of which were presented to the Veterinary Teaching Hospital at the University of Las Palmas de Gran Canaria. The research was conducted in a region with a high prevalence of the disease [25] between September 2021 and July 2023. Diagnosis was confirmed using an immunocromatographic test kit for detection of *D. immitis* antigens (Uranotest Dirofilaria, Urano Vet SL, Barcelona, Spain), in accordance with the manufacturer’s instructions. In addition, the modified Knott test was performed to assess the presence of circulating microfilariae.

A detailed record was maintained for each dog, including breed, sex, and age. All dogs underwent a complete physical examination and clinical history review. Animals exhibiting one or more clinical signs commonly associated with heartworm disease—such as dyspnea, coughing, exercise intolerance, weakness, weight loss, syncope, or signs of right-sided congestive heart failure (e.g., ascites, jugular venous distension, and hepatomegaly)—were classified as symptomatic.

Inclusion criteria required that dogs had not received previous heartworm prophylaxis or undergone any previous treatment for *D. immitis*. Informed consent was obtained from all owners prior to participation. Dogs were excluded if they presented with concurrent cardiopulmonary conditions (e.g., left-sided heart disease, dilated cardiomyopathy, congenital abnormalities, chronic respiratory diseases), as determined by clinical evaluation, medical history, and further diagnostic testing (e.g., thoracic X-rays, blood work). Dogs with abnormal serum creatinine or blood urea nitrogen (BUN) concentrations, evidence of anemia, or systemic hypertension were also excluded to minimize potential interference with NT-proBNP concentrations [27, 28].

Dogs received adulticide therapy following the general principles outlined in the guidelines established by international heartworm societies, particularly the American Heartworm Society and European Society of Dirofilariosis and Angiostrongylosis, while incorporating modifications based on recently published clinical protocols [29–31]. Specifically, treatment began on day 0 with a 4-week course of doxycycline (10 mg/kg, BID) to target *Wolbachia pipientis* endosymbionts. Concurrently, a monthly oral combination of ivermectin (≥ 6 µg/kg) and pyrantel pamoate (≥ 5 mg/kg) was administered for macrocyclic lactone-based heartworm prevention and larvae elimination. Following the protocol described by Carretón et al. [31], a three-dose melarsomine regimen was initiated with a single injection on day 30, followed by two injections on days 60 and 61 (2.5 mg/kg each). Dogs were reassessed on day 90, and discharge was granted in the absence of abnormalities, such as adult heartworms detected via echocardiography, radiographic changes, or cardiorespiratory signs. A final antigen test was conducted on day 270 (6 months post-discharge) to confirm the effectiveness of the adulticide protocol. Physical activity was restricted throughout the treatment, with particular emphasis on the period between the first melarsomine injection and discharge.

### Echocardiographic evaluation

All dogs underwent echocardiographic evaluation on days 0, 30, 60, and 90, using a system equipped with spectral and color Doppler and multifrequency transducers (2.5–10 MHz, Vivid Iq^®^, General Electric, Boston, MA, USA). Examinations were performed on conscious dogs positioned in right and left lateral recumbency under electrocardiographic monitoring. All echocardiographic measurements were performed by a single operator. The presence or absence of PH was assessed following the guidelines of the American College of Veterinary Internal Medicine (ACVIM) [1]. Tricuspid regurgitation systolic flow was assessed using color-flow Doppler superimposed on real-time 2D images, and continuous-wave spectral Doppler was used to measure tricuspid regurgitant velocity (TRV), as previously described [22].

The right pulmonary artery distensibility index (RPAD index) was also evaluated owing to its relevance in heartworm disease [11, 32–34]. Other echocardiographic parameters measured included tricuspid regurgitation pressure gradient (TRPG), pulmonary trunk to aorta ratio (PT:Ao), global tissue Doppler imaging (G-TDI) index, tricuspid annular plane systolic excursion (TAPSE), pulmonary vein to pulmonary artery ratio (PV:PA), right ventricular acceleration time (AT), right ventricular ejection time (ET), AT:ET ratio, right atrial area index (RAAi), right ventricular end-diastolic area index (RVEDAi), and right ventricular to right atrial pressure gradient (RV:RA PG). The modified Bernoulli equation (pressure gradient [mmHg] = 4 × velocity^2^ [m/s^2^]) was applied to TRV to calculate RV:RA PG. An RV:RA PG > 40 mmHg, in the absence of pulmonary valve stenosis or right ventricular outflow tract obstruction, was considered diagnostic of PH [22, 26].

For RPAD index and PV:PA ratio measurements, dogs were positioned in right lateral recumbency, and the transducer was placed in the third intercostal space with the beam directed caudally and dorsally. Pulmonary artery measurements were obtained at maximal systolic and minimal diastolic dimensions following previously established methodologies [35]. TRPG, PT:Ao ratio, G-TDI, TAPSE, AT, ET, AT:ET ratio, RAAi, and RVEDAi were measured using apical four-chamber and left cranial transverse views [36].

Each echocardiographic parameter was recorded over three consecutive cardiac cycles. Dogs were classified as having moderate-to-severe PH if they met any of the following echocardiographic criteria, according to the guidelines of the American College of Veterinary Internal Medicine (ACVIM) [1]: RPAD index < 29.5%, tricuspid regurgitation systolic flow > 3.4 m/s, TRPG < 30 mmHg, PA:Ao ratio > 1.05–1.23, AT > 5.50 ± 0.31 ms, ET > 10.6 ± 0.14 ms, AT:ET ratio < 0.30, G-TDI > 11.8 ± 8.50, RVEDAi > 4.9–10.9 cm^2^/m^2^, RAAi > 4.2–10.2 cm^2^/m^2^, and TAPSE > 4.78–7.64 mm (Table 1).

**Table 1:** Echocardiographic parameters measured in dogs with and without pulmonary hypertension on days 0, 30, 60 and 90

Parameter	Day 0	Day 30	Day 60	Day 90
Dogs with pulmonary hypertension (*n* = 31)				
RPAD index (%)	22.3 (19.8–23.7)	25.5 (23.8–26.1)	30.8 (28.7–31.2)	34.6 (32.8–35.6)
TRPG (mmHg)	111.8 (78.0–128.5)	68.7 (64.5–73.2)	46.1 (45.0–52.0)	27.2 (25.2–31.5)
PT:Ao ratio	1.74 (1.62–1.78)	1.54 (1.50–1.60)	1.41 (1.36–1.47)	1.29 (1.26–1.35)
G-TDI (m/s)	7.6 (7.3–8.4)	7.9 (7.5–8.5)	8.0 (7.9–8.7)	8.4 (8.2–8.9)
TAPSE (cm)	1.02 (0.95–1.15)	1.21 (1.15–1.25)	1.38 (1.32–1.45)	1.59 (1.50–1.65)
PV:PA	0.68 (0.65–0.78)	0.81 (0.78–0.90)	0.91 (0.85–0.99)	0.97 (0.96–1.05)
AT (ms)	59 (55–70)	78 (70–90)	98 (90–110)	120 (115–135)
ET (ms)	211 (195–225)	225 (210–240)	236 (220–250)	250 (230–265)
AT:ET	0.27 (0.28–0.34)	0.34 (0.32–0.40)	0.41 (0.39–0.47)	0.49 (0.45–0.54)
RAAi (cm^2^/m^2^)	18.7 (16.0–20.0)	15.9 (14.8–18.5)	14.1 (13.5–17.0)	13.5 (12.0–15.5)
RVEDAi (cm^2^/m^2^)	20.2 (17.0–21.5)	16.5 (15.9–19.8)	15.1 (14.4–18.3)	14.3 (13.2–17.0)
RV:RA PG (mmHg)	8.7 (7.0–9.3)	6.5 (5.2–6.8)	4.2 (3.8–5.2)	2.9 (2.5–3.7)
Dogs without pulmonary hypertension (*n* = 59)				
RPAD index (%)	36.1 (34.0–36.5)	35.2 (34.8–37.1)	35.8 (35.4–37.5)	36.3 (36.0–38.2)
TRPG (mmHg)	21.3 (20.5–27.0)	20.9 (19.8–25.0)	19.6 (18.0–23.0)	19.2 (17.2–22.0)
PT:Ao ratio	1.17 (1.10–1.20)	1.12 (1.05–1.15)	1.08 (1.00–1.10)	1.03 (0.95–1.05)
G-TDI (m/s)	8.0 (7.8–8.4)	8.5 (8.0–8.6)	8.7 (8.2–8.8)	8.6 (8.3–8.9)
TAPSE (cm)	1.58 (1.50–1.70)	1.66 (1.55–1.75)	1.72 (1.58–1.78)	1.75 (1.60–1.80)
PV:PA	0.97 (0.95–1.05)	1.08 (1.00–1.10)	1.06 (1.03–1.13)	1.11 (1.05–1.15)
AT (ms)	115 (100–120)	119 (110–130)	135 (120–140)	138 (125–145)
ET (ms)	219 (210–230)	228 (220–240)	235 (230–250)	243 (235–255)
AT:ET	0.48 (0.45–0.55)	0.51 (0.48–0.56)	0.57 (0.50–0.58)	0.54 (0.51–0.59)
RAAi (cm^2^/m^2^)	13.9 (12.0–15.5)	12.8 (11.5–14.2)	11.9 (11.0–13.8)	11.5 (10.5–13.0)
RVEDAi (cm^2^/m^2^)	15.4 (13.0–16.0)	13.6 (12.5–15.5)	13.4 (12.0–15.0)	12.7 (11.5–14.5)
RV:RA PG (mmHg)	3.1 (2.2–3.4)	2.4 (2.1–3.1)	2.2 (2.0–2.8)	2.1 (1.8–2.6)

RPAD index, right pulmonary artery distensibility index; TRPG, tricuspid regurgitation pressure gradient; PT:Ao ratio, pulmonary trunk to aorta ratio; G-TDI, global tissue Doppler imaging; TAPSE, tricuspid annular plane systolic excursion; PV:PA, pulmonary vein to pulmonary artery ratio; AT, right ventricular acceleration time; ET, right ventricular ejection time; RAAi, right atrial area index; RVEDAi, right ventricular end-diastolic area index; RV:RA PG, right ventricular to right atrial pressure gradient

Furthermore, on day 0, parasite burden was assessed by echocardiography using a previously established scoring system [11], categorizing relative parasite loads from 1 to 4, with scores 1–2 considered low (parasites not visible or only a few echoes in the distal part of the right pulmonary artery) and scores 3–4 considered high (worm echoes occupying the right pulmonary artery or extended to the main pulmonary artery).

### Systemic blood pressure determination

Blood pressure was measured on days 0, 30, 60, and 90, using a high-definition oscillometric device (Vet HDO Monitor^®^, S + B medVet GmbH, Babenhausen, Germany). The cuff size was selected on the basis of the diameter of the forelimb or, alternatively, the tail, depending on the dog. The final blood pressure value was calculated as the mean of three consecutive measurements taken at 5-min intervals. Dogs exhibiting signs of stress likely to affect blood pressure readings were allowed time to rest, adjust, or calm down. Systemic arterial hypertension was defined as sustained elevations in systolic blood pressure (SBP) exceeding 160 mmHg in accordance with established consensus guidelines [37]. The results were not evaluated in the statistical study, since only normotensive dogs were included in the study and this test was only performed to exclude the presence of hypertensive dogs from the study.

### Serum analysis

For NT-proBNP analysis, a minimum of 2 mL of blood was collected from the cephalic vein of each dog into serum tubes. Samples were collected on days 0, 30, 60, and 90, centrifuged at 1432 *g* for 10 min and analyzed within 2 h. NT-proBNP concentrations were measured using the VCHECK immunochromatographic analyzer (Bionote, Big Lake, MN, USA), which has been validated for use in dogs [38]. According to the manufacturer, reference values for healthy dogs are < 900 pmol/L. Renal parameters (creatinine and BUN) were also evaluated to identify any renal impairment that could affect NT-proBNP levels [27].

### Statistical analyses

Statistical analyses were performed using SPSS^®^ Statistics 25.0 (IBM, New York, USA). Categorical variables (e.g., breed, sex, clinical signs, microfilariae presence, parasite burden, PH status) were expressed as frequencies and percentages. Continuous variables (e.g., age, NT-proBNP concentrations) were reported as means ± standard deviation, and medians and interquartile range. Comparisons of continuous variables were made using Mann–Whitney or Kruskal–Wallis tests for nonparametric data and *t*-tests or analysis of variance (ANOVA) for parametric data, with normality assessed via the Shapiro–Wilk test. Differences in categorical variables were assessed using Pearson’s Chi-squared test. Post hoc pairwise comparisons were performed with Bonferroni correction when applicable. Effect sizes were calculated using Cramer’s V for categorical variables and Cohen’s D for continuous variables. Receiver operating characteristic (ROC) curve analysis was used to determine the optimal NT-proBNP cut-off value for predicting an RPAD index < 29.5%, with the Youden index applied to maximize sensitivity and specificity. A *P*-value < 0.05 was considered statistically significant.

## Results

The group of dogs with PH on day 0 (*n* = 31) included 16 mixed-breed dogs, 3 Andalusian Terriers, 3 Labrador Retrievers, 2 American Pit Bull Terriers, and 1 each of the following breeds: Pug, Smooth Fox Terrier, Canary Hound, Canary Dog, French Bulldog, Pointer, and Boxer. The group of dogs without PH on day 0 (*n* = 59) consisted of 42 mixed-breed dogs, 4 Canary Dogs, 4 German Shepherds, 2 Canary Hounds, 2 Majorero Dogs, and 1 each of the following breeds: Rottweiler, Miniature Pinscher, English Water Spaniel, Xoloitzcuintle, and American Pit Bull Terrier.

NT-proBNP was pathologically increased in 23 dogs on day 0. Of these, 19 dogs (61.3%) were classified within the PH group, whereas 4 dogs (6.8%) belonged to the normotensive group. The results showed that sex, breed, and renal values were not significantly different between dogs with/without PH. However, dogs with PH were significantly older (8.22 ± 3.09 years) than those without PH (5.32 ± 2.89 years) (*U* [456.5], *P* 0.002, *d* 0.898). Analysis of epidemiological variables in relation to NT-proBNP concentrations revealed significant differences in biomarker levels only with respect to the age of the dogs studied (*r* = 0.29, *P* = 0.007).

At baseline (day 0), the presence of circulating microfilariae did not significantly influence NT-proBNP concentrations (*r*_s_ = 0.077, *P* = 0.476). Microfilariemic dogs (*n* = 48) had a mean NT-proBNP concentration of 1212.66 ± 1392.79 pmol/L, compared with 932.68 ± 913.94 pmol/L in amicrofilariemic dogs (*n* = 42). In contrast, parasite burden was significantly associated with NT-proBNP levels. A moderate positive correlation was observed between worm burden and NT-proBNP concentrations (*r*_*s*_ = 0.530, *P* < 0.05). When parasite burden was categorized as low (scores 1–2; *n* = 40) or high (scores 3–4; *n* = 50), dogs in the high-burden group exhibited significantly higher NT-proBNP concentrations (1478.79 ± 1526.73 pmol/L) than those with a low burden (634.77 ± 283.16 pmol/L) (*r* _s_= 0.370, *P* < 0.001*).*

A significant positive correlation was found between NT-proBNP concentrations and the presence and severity of PH. Both Pearson’s (*r*_*(123)*_ = 0.576, *P* < 0.05) and Spearman’s (*r*_*s*_ = 0.509, *P* < 0.05) correlation coefficients indicated that elevated biomarker levels were associated with increased severity PH. Moreover, dogs classified as having moderate-to-severe PH on day 0 had significantly higher NT-proBNP concentrations (2038 ± 1671.36 pmol/L) compared with dogs without PH (583.27 ± 185.18 pmol/L) (*P* < 0.001).

At baseline, 44.44% (*n* = 40) of the dogs showed clinical signs. Dogs presenting with symptoms such as dyspnea, coughing, and exercise intolerance had significantly higher NT-proBNP levels (1696.69 ± 1591.62 pmol/L) than asymptomatic dogs (594.69 ± 248.72 pmol/L). This association was supported by Pearson’s (*r*_*(123)*_ = 0.456, *P* < 0.05) and Spearman’s (*r*_*s*_ = 0.473, *P* < 0.05) correlations.

**Fig. 1:**
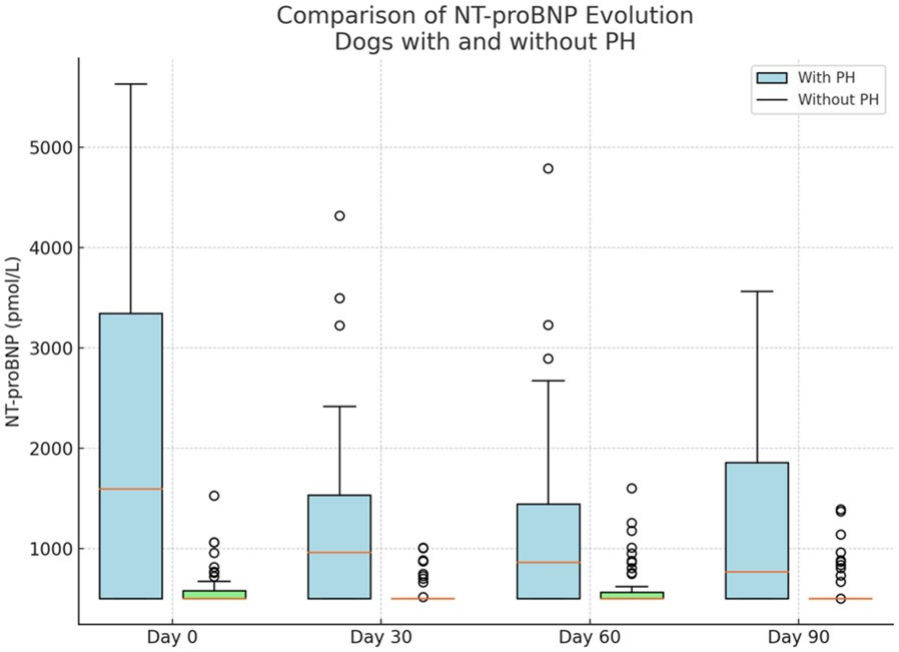
Box-and-whisker plots showing the distribution of NT-proBNP concentrations (pmol/L) in dogs with pulmonary hypertension (PH) and without PH at days 0, 30, 60, and 90. Blue boxes represent dogs diagnosed with PH, and green boxes represent those without PH. The horizontal line within each box indicates the median; the box spans the interquartile range (IQR), and whiskers extend to 1.5 × IQR. Outliers are shown as individual points. Dogs with PH exhibited consistently higher NT-proBNP values across all time points

Significant changes in NT-proBNP concentrations were observed over the evaluation period (days 0, 30, 60, and 90), particularly in the PH group. Among dogs with PH, a progressive decrease in NT-proBNP values was recorded: 1665.45 ± 1512.97 pmol/L on day 30, 1543.23 ± 1428.69 pmol/L on day 60, and 1462.14 ± 1355.46 pmol/L on day 90. In contrast, NT-proBNP concentrations in the non-PH group remained stable, with values of 512.98 ± 176.43 pmol/L, 501.36 ± 169.85 pmol/L, and 489.14 ± 160.72 pmol/L on days 30, 60, and 90, respectively (Fig. 1).

Repeated-measures ANOVA did not reveal a significant overall trend over time (PH group: *F* = 1.16, *P* = 0.331; non-PH group: *F* = 0.36, *P* = 0.779). In the group with PH, pairwise comparisons revealed a significant decrease in NT-proBNP levels between day 0 and day 90 (*U*_(18)_ = 3.13, *Z* = 1.77, *P* = 0.016). No significant differences were observed between day 0 and day 30 (*U*_(18)_ = 3.14, *Z* = 1.58, *P* = 0.446), day 0 and day 60 (*U*_(18)_ = 3.42, *Z* = 0.61, *P* = 0.627), or day 30 and day 90 (*U*_(18)_ = 3.26, *Z* = 0.23, *P* = 0.345). However, a significant reduction was found between day 30 and day 60 (*U*_(18)_ = 3.01, *Z* = 0.99, *P* = 0.022). In the group without PH, none of the pairwise comparisons reached statistical significance (all *P* > 0.05), confirming the stability of NT-proBNP concentrations in these dogs.

Using a cutoff of 900 pmol/L, NT-proBNP yielded a sensitivity of 61.29% and a specificity of 93.85% for detecting PH. For identifying moderate-to-severe PH, a threshold of ≥ 1524.8 pmol/L provided a sensitivity of 99% and specificity of 87% (AUC = 0.95; *P* < 0.05). This cutoff was validated by the Youden index (0.86), which confirmed optimal diagnostic performance.

## Discussion

The results of this study reinforce the usefulness of NT-proBNP as a biomarker for assessing the presence of PH in dogs infected with *D. immitis*, as shown in a previous study [24]. The association between NT-proBNP and precapillary PH can be explained by increased pulmonary arterial pressure and right ventricular overload, which lead to the release of this biomarker in response to myocardial wall stress [17]. Although the primary source of BNP is left ventricular myocardial stress, the right ventricle is also a source of BNP, and other studies have reported elevated NT-proBNP levels in canine diseases causing precapillary hypertension [21, 22, 28].

A positive correlation between parasite burden and NT-proBNP levels was also observed, suggesting that a higher number of adult parasites may exacerbate pulmonary vascular remodeling and right ventricular dysfunction, promoting the development and progression of PH, as previously suggested by other authors [39–41]. However, these results differ from those previously obtained by the same research team in a similar study [24]. The reason is unclear and may be due to confounding factors, such as the lifestyle habits of the infected dogs. For example, it is known that intense physical exercise worsens vascular pathology regardless of the parasite burden [42–44] and, in this study, such data were unavailable, as many of the animals had been recently adopted from shelters.

NT-proBNP values were significantly higher in older dogs. These results are consistent with previous reports [24], likely because in endemic areas older dogs are more prone to chronic infections and therefore more severe and long-standing vascular damage, leading to PH more frequently [25, 45]. Likewise, symptomatic dogs showed significantly higher NT-proBNP concentrations than asymptomatic ones, as in previous studies [24, 46], probably due to being in more advanced and chronic stages of the disease [2, 46]. Other studies emphasize the importance of including PH in the differential diagnosis of elevated cardiac biomarkers in dogs with respiratory signs, highlighting the role of NT-proBNP in both screening and monitoring disease progression [22]. This supports NT-proBNP as a severity marker in dogs with PH, as its elevation is associated with symptoms such as dyspnea, coughing, and exercise intolerance [6, 28]. However, the presence of elevated NT-proBNP in some asymptomatic dogs suggests that it may also detect subclinical changes, making it valuable for early identification of PH in its initial stages [13].

Another finding was the absence of a statistically significant relationship between the presence of microfilariae and NT-proBNP levels. This result aligns with previous studies [24, 33] indicating that microfilariae have a minor impact on pulmonary hemodynamics compared with adult worm burden.

Receiver operating characteristic (ROC) curve analysis identified an NT-proBNP cutoff value of 1524.8 pmol/L for predicting moderate-to-severe PH, with 99% sensitivity and 87% specificity. This cutoff differs from the one obtained previously in a similar study, which was established at 1178.75 pmol/L, but with lower sensitivity and higher specificity (64.3% and 95.5%, respectively) [24]. It is also considerably higher than the value reported by Kellihan et al. [28], who found an NT-proBNP cutoff of 900 pmol/L with 91.7% sensitivity and 62.5% specificity in 12 dogs with precapillary PH. The high sensitivity and specificity obtained in this study indicate that NT-proBNP may be a reliable marker for detecting PH in dogs with *D. immitis*, facilitating more accurate diagnosis and reducing operator dependence in echocardiographic assessments [7].

In addition, this study evaluated the evolution of this biomarker throughout adulticide treatment. A gradual decrease in mean NT-proBNP values was observed at each serial measurement, with a statistically significant reduction in biomarker concentrations between day 30 and day 60. While the first dose of melarsomine has been shown to induce the death of approximately 51.7% of adult heartworms over the subsequent weeks [47], it is important to note that neither complete parasite elimination nor a rapid resolution of pulmonary hypertension is expected at this stage. In fact, the onset of pulmonary thromboembolism resulting from worm death may transiently exacerbate vascular pathology [2, 3]. Nonetheless, the observed NT-proBNP decline may reflect early hemodynamic stabilization resulting from several overlapping factors. First, the reduction in adult worm load may already alleviate some degree of mechanical obstruction and shear stress, contributing to decreased release of vasoconstrictive mediators such as endothelin-1 and parasitic metabolic byproducts [3, 44, 48]. Second, prior elimination of *Wolbachia pipientis* through doxycycline therapy has been reported to reduce pulmonary inflammation and endarteritis [49], thereby mitigating vascular injury and immune-mediated responses to worm death. Together, these effects may have contributed to a modest improvement in right ventricular load and myocardial stress, even in the continued presence of pulmonary hypertension. Thus, while echocardiographic indicators of pulmonary hypertension may not show immediate improvement, the decline in NT-proBNP likely reflects the early composite impact of reduced parasite-induced inflammation, partial worm clearance, and improved vascular homeostasis.

NT-proBNP values reached their lowest levels in PH dogs at the end of treatment, although they remained within pathological ranges. This finding agrees with previous reports stating that pulmonary endarteritis is not reversible, and once the parasites are eliminated, PH will persist [34]. However, according to those same authors, it may still be too early to determine the irreversibility of PH, as some worms may still be dying, and arterial inflammation may take time to subside [34, 50, 51], so it would be interesting to evaluate this biomarker in these animals at a later timepoint.

However, dogs without PH did not show significant alterations in NT-proBNP levels during adulticide treatment. For all these reasons, NT-proBNP is proposed as a highly useful serological marker for monitoring adulticide treatment in infected dogs [5].

In conclusion, the findings of this study strengthen the usefulness of NT-proBNP as a biomarker to detect, assess severity, and monitor treatment response in PH associated with canine heartworm disease. Given its high predictive value and clinical applicability, incorporating NT-proBNP into diagnostic protocols could improve early detection and management of this condition, ultimately enhancing patient outcomes.

## Data Availability

No datasets were generated or analyzed during the current study.

## Abbreviations

PH: Pulmonary hypertension
NT-proBNP: N-terminal pro B-type natriuretic peptide
